# Large Extracellular Loop of Tetraspanin as a Potential Vaccine Candidate for Filariasis

**DOI:** 10.1371/journal.pone.0077394

**Published:** 2013-10-11

**Authors:** Gajalakshmi Dakshinamoorthy, Gnanasekar Munirathinam, Kristen Stoicescu, Maryada Venkatarami Reddy, Ramaswamy Kalyanasundaram

**Affiliations:** 1 Department of Biomedical Sciences, University of Illinois College of Medicine at Rockford, Rockford, Illinois, United States of America; 2 Department of Biochemistry, Mahatma Gandhi Institute of Medical Sciences, Sevagram, Maharashtra, India; Queensland Institute of Medical Research, Australia

## Abstract

Lymphatic filariasis affects nearly 120 million people worldwide and mass preventive chemotherapy is currently used as a strategy to control this infection. This has substantially reduced the incidence of the infection in several parts of the world. However, a prophylactic vaccine would be more effective in preventing future infections and will supplement the mass chemotherapy efforts. Unfortunately, there is no licensed vaccine available currently to prevent this infection. Molecules expressed on the surface of the parasite are potential candidates for vaccine development as they are exposed to the host immune system. In this study we show that the large extracellular loop of tetraspanin (TSP LEL), a protein expressed on the cuticle of *Brugia malayi* and *Wuchereria bancrofti* is a potential vaccine candidate. Our results showed that *Bm*TSP LEL is expressed on the surface of *B. malayi* infective third stage larvae (L3) and sera from human subjects who are putatively immune to lymphatic filariasis carry high titer of IgG1 and IgG3 antibodies against *Bm*TSP LEL and *Wb*TSP LEL. We also showed that these antibodies in the sera of human subjects can participate in the killing of *B. malayi* L3 in an antibody dependent cell-mediated cytotoxicity mechanism. Vaccination trials in mice showed that close to 64% protection were achieved against challenge infections with *B. malayi* L3. Immunized animals showed high titer of anti-*Wb*TSP LEL IgG1, IgG2a and IgG2b antibodies in the sera and IFN-γ secreting cells in the spleen. *Onchocerca volvulus* another filarial parasite also expresses TSP LEL. Cross-reactivity studies showed that IgG1 antibody in the sera of endemic normal subjects, recognize *Ov*TSP LEL. Similarly, anti-*Ov*TSP LEL antibodies in the sera of subjects who are immune to *O. volvulus* were also shown to cross-react with r*Wb*TSP LEL and r*Bm*TSP LEL. These findings thus suggested that rTSP LEL can be developed as a potential vaccine candidate against multiple filarial infections.

## Introduction

Lymphatic filariasis (LF) affects 120 million people in 72 countries and more than 1.2 billion people are at the risk of acquiring the infections [1]. The disease is transmitted by mosquitoes carrying any of three parasitic nematodes: *Wuchereria bancrofti*, *Brugia malayi*, and *Brugia timori*. In the tropical and sub tropical regions of the world filarial infections are a major public health and socio-economic problem [2,3]. Mass drug administration is currently used as an approach to control this infection in endemic areas [4]. However, an effective prophylactic vaccine against lymphatic filariasis coupled with effective vector control measures can supplement the mass drug administration approach and speed up the effort to eliminate this infection in the endemic regions. Several potential vaccine candidates have been identified and evaluated from different laboratories [5-9], yet there is no effective vaccine available against this disease. This may be largely due to the complex life cycle stages of the parasites and the varied immune responses developed by the host to each antigen.

Lymphatic filarial parasites are notorious for evading the host immune responses by molecular mimicry and active modulation of the host immune responses [10]. Tetraspanin (TSP) family of proteins is one among the parasite antigens that plays an important role in host immunomodulation. TSPs are membrane-spanning proteins containing four transmembrane domains, three short intracellular domains, and two extracellular loops, EC1 and EC2 [11]. TSPs are abundantly expressed on the surface or body wall of several helminth parasites including *S. mansoni* [12], *S. japonicum* [13] and in the nematode *C. elegans* [14]. TSP is believed to play important roles in signal transduction, cell proliferation, adhesion, migration, fusion and host-parasite interactions [15]. Among the various TSPs reported, TSP-2 identified from *Schistosoma mansoni* (*Sm*TSP-2) is shown to be involved in the formation of tegument [13] which is critically important for the parasite’s survival in the host [16]. Since TSPs are expressed on the surface, they are easy targets for immune attack by host antibodies and cells [17]. Especially, the extracellular loops of TSP are more amenable to the host immune system. Given its critical role in the formation of tegument and survival of the parasite in the host, TSP has been a target for developing vaccine against *S. mansoni* [18-20]. Especially, the large extracellular loop (LEL) of *Sm*TSP-2 was shown to be highly effective as a vaccine immunogen conferring high levels of protection in mice against challenge infections [12]. Previously we reported the presence of *Sm*TSP-2 homologue in *B. malayi* parasites [21]. Since TSP-LEL appears to be a good vaccine candidate, in the present study we cloned *tsp lel* from lymphatic filarial parasites (*W. bancrofti* and *B. malayi*). We then expressed the recombinant *B. malayi* TSP LEL (r*Bm*TSP LEL) and *W. bancrofti* TSP LEL (r*Wb*TSP LEL) proteins and evaluated its vaccine potential in human and in mice.

Developing a single global vaccine that is effective against multiple infections is an attractive strategy for endemic regions where mixed infections are common. Administering vaccines separately for diseases require an increased number of visits, labor, side effects, and costs not to mention the various regulatory approvals needed to develop separate new vaccines. Generation of broadly protective “universal” vaccines restricted to species or groups of closely related pathogens or even cross-family or -kingdom vaccines might be useful to overcome some of the barriers in vaccine development [22]. Very few studies have attempted to identify these cross-species protective antigens. Among the eight filarial species that parasitize humans, lymphatic filariasis caused by *W. bancrofti* and *B. malayi* and onchocerciasis caused by *Onchocerca volvulus* are more common and occur as co-infection in several endemic regions [23,24]. These three parasites are also the major targets for vaccine development by a number of laboratories [23,25-27]. In this report we show that homologue of TSP-2 is expressed in *O. volvulus* and show significant sequence similarity to *Bm*TSP-2 and *Wb*TSP-2. Therefore, in this study we have also attempted to evaluate the cross-protection potential of rTSP LEL from LF parasites against onchocerciasis.

## Materials and Methods

### Ethics Statement

Use of human subjects in this study was approved by the Institutional Review Board (IRB) of the University of Illinois, College of Medicine at Rockford; the New York Blood Center’s IRB and by an NIH accredited Institutional Review Board of the Medical Research Council Kumba, Cameroon and the IRB committee of Mahatma Gandhi Institute of Medical Sciences, Sevagram, Maharashtra, India. Informed written consent in native language was obtained from all the subjects before collecting the samples. Humane use of animals was performed in this study according to the guidelines for the care and use of laboratory animals and with the rules formulated under the Animal Welfare Act by the U.S. Department of Agriculture. The protocol was approved by The IACUC Committee of the University of Illinois, College of Medicine at Rockford and performed at a facility accredited by AAALAC and USDA.

### Parasites

*Brugia malayi* infective third stage larvae (L3) were obtained from the NIAID/NIH Filariasis Research Reagent Resource Center (FR3) at the University of Georgia, Athens, GA.

### Human sera Samples

Blood samples were collected after taking informed consent from clinically diagnosed filarial patients and from healthy adult individuals residing in Sevagram and surrounding villages in Maharashtra State, which are non-coastal endemic areas for nocturnally periodic *W. bancrofti* infection. Samples were also collected from volunteers who live in areas that are non endemic for filariasis. Parasitological examination of all individuals was done by detection of microfilariae in night blood smears. The presence of mf was further confirmed by membrane (Millipore-5m filters) filtration of 1.0 ml of heparinized venous blood [28]. The presence of circulating antigen was detected using a *Wb*SXP based enzyme-linked immunosorbent assay (ELISA) [29]. About 10 ml of blood samples (5 ml in heparinized tubes) were collected from the following clinical groups of subjects (a) Endemic normal (EN) subjects, these are individuals who are asymptomatic and non-microfilaraemic residing in Sevagram (b) Asymptomtic microfilaraemic subjects (MF) were classified based on the presence of circulating mf in blood. (c) Chronic Pathology (CP) patients include those subjects who exhibit lymph edema and other chronic clinical symptoms of filariasis. These CP patients did not carry circulating mf and (d) Non-endemic normal (NEN) subjects are healthy individuals who live in a non-endemic country and have no circulating parasites or antibodies in their sera and show no evidence of any clinical lymphatic filarial disease. Sera were separated from these blood samples and were stored at - 80°C until use. PBMC proliferation was performed using freshly collected blood samples from EN, MF and CP individuals.

Sera samples from onchocerciasis immune (PI) and onchocerciasis infected individuals (INF) were collected from residents of 5 villages around Kumba, Cameroon, a hyperendemic area after obtaining the informed consent by signing or thumb printing a consent form after reading it or after the content of the form was read and/or explained to them. These villages were Marumba I, Marumba II, Boa Bakundu, Bombanda, and Bombele. All participants were born or had resided for more than 10 years in the villages. The standard skin snip test for detection of microfilariae (mf) was performed on each subject and clinical symptoms of onchocerciasis were recorded. The averages of the mf counts of four skin snips taken from each individual were used in estimating the individual skin mf densities. None of the subjects had received ivermectin treatment prior to the collection of blood. Serum samples of 20 putatively immune (PI) subjects of onchocerciasis were studied. These individuals had no signs or history of onchocerciasis and were parasitologically negative during at least a two-year follow-up survey employing the standard skin snip test and a polymerase chain reaction (PCR) assay. Age, sex matched infected individuals were used for comparison as described before [30,31] .

### Cloning of tsp from three filarial parasites

Transcripts encoding TSP LEL proteins were amplified from *W. bancrofti*, *B. malayi* and *O. volvulus* L3 cDNA libraries. Primers were designed from the genebank sequence (Accession number EF397425.1) of *B. malayi* tetraspanin LEL domain. The same set of primers were used to amplify *tsp lel* from the cDNA libraries of all the three parasites; Forward primer sequence with BamHI restriction sites 5′-CG**GGATCC**CGGCAAGGATCAATTTAAAA-3′ and Reverse primer sequence with EcoRI 5′-CG**GAATTC**TCAATCTTTTTGAGATGAAT-3′. PCR was performed with denaturation at 95°C, annealing at 50°C, and extension at 75°C. Five different PCR clones from each parasite species were sent out for sequencing at the DNA sequencing facility at UIC, Chicago. The results showed no difference in the sequences between the PCR clones. A multiple sequence alignment of helminth TSP LEL was then performed using ClustalW.

### Expression and purification of rTSP LEL domain

*tsp lel* amplified from L3 cDNA libraries (*W. bancrofti*, *B. malayi* and *O. volvulus*) were cloned into pRSETA expression vector and transformed into BL21(DE3) pLysS *E. coli* expression host. *E. coli* cultures with *pRSETA tsp* plasmid was inoculated in 500ml of LB broth and incubated for 3 hours until the log phase of bacterial growth was reached (absorbance OD 0.6 at 600nm). 1 mM of isopropyl-beta-D-thiogalactopyranoside [IPTG] was added to the culture to induce the proteins expressions and incubated for an additional 3 hours. Recombinant *Wb*TSP LEL, *Bm*TSP LEL and *Ov*TSP LEL expressed as His-tag proteins were purified using IMAC column. SDS PAGE was run to check the purity of the recombinant proteins. Immunoblot was performed by probing with anti penta-His antibodies (Qiagen, Valencia, CA). Endotoxin was removed from the recombinant proteins using High Capacity Endotoxin removal resin (Thermo Fisher Scientific, Rockford, IL). Levels of endotoxin in the final recombinant protein preparation were confirmed by LAL assay (Gen Script, Piscataway, NJ) and were found to be below 4 EU/ml.

### Immunization of mice

Six weeks old male Balb/c mice purchased from Charles River laboratory (Wilmington, MA) were divided into three groups with five mice in each group. Mice were immunized four times subcutaneously (s.c) with 15μg of recombinant tetraspanin proteins (r*Wb*TSP LEL or r*Bm*TSP LEL) in 100μl volume combined with 50μl of Imject alum (Thermo Fisher Scientific) given at 2 weeks interval. Control group of animals received only alum. Blood was collected from the retro-orbital space of each mouse two weeks after the final dose of immunization. This experiment was repeated two times. 

### Immunofluorescence assay to evaluate the presence of TSP LEL on the surface of *B. malayi* L3

Presence of TSP LEL on the surface of infective larvae of *B. malayi* was analyzed by an immunofluorescence whole mount assay [32]. About 5-10 L3 were incubated with 1% BSA, 0.1% Triton X-100 in phosphate buffer saline (PBS) for two hours at 4°C under agitation in the microcentrifuge tube. Pooled mouse anti-*Bm*TSP LEL sera (optimum dilution was found to be 1:25) diluted in 100µl volumes of blocking buffer was added to L3 and incubated at room temperature for 2 h. After washing with PBS, the parasites were incubated at RT for 1 h with anti-mouse IgG conjugated with fluorescein isothiocyanate (FITC, purchased from Thermo Fisher Scientific) at 1:1000 dilutions. After washing with PBS, the parasites were placed on to a glass slide and mounted with fluorophore mounting medium. After placing a cover slip, the edges were sealed and observed under a fluorescence microscope (Olympus, Center valley, PA) using 10X and 60X objective lens. Sera from mice immunized with alum or anti-mouse IgG FITC alone were used as negative controls (data not shown).

### Analysis of total anti-TSP LEL IgG antibodies in the sera samples

IgG antibodies against r*Wb*TSP LEL, r*Bm*TSP LEL or r*Ov*TSP LEL were evaluated in the sera of different filarial groups of subjects by indirect ELISA [33]. Briefly, wells of a 96 well microtiter ELISA plates were coated with 100 ng/well of recombinant proteins (r*Wb*TSP LEL, r*Bm*TSP LEL or r*Ov*TSP LEL) in 0.05M carbonate-bicarbonate buffer, pH 9.6. After blocking the wells with 3% BSA in PBS-Tween 20 (PBS-T, 0.05%), 100µl of sera samples (1:100 diluted in PBS-T) from different groups of subjects with filarial infections (MF, CP, EN and NEN) were added to each well. Horse radish peroxidase-labeled Goat anti-human IgG was used as the secondary antibody. The color was developed using OPD substrate and absorbance was read at 450 nm in the ELISA reader (BioRad, Hercules, CA). Antigen-specific isotype of IgG antibodies present in the sera of different filarial groups were evaluated by indirect ELISA [34]. Biotinylated anti-human IgG1 (1:1000), IgG2 (1:15000), IgG3 (1:1000) and IgG4 (1:15000) isotypes (Sigma Aldrich, St. Louis, MO) were used as the secondary antibodies. Levels of anti-TSP LEL IgG and IgG isotype antibodies (Biolegend, San Diego, CA) were similarly measured in the sera of mice immunized with rTSP LEL proteins.

Levels of cross-reactive IgG1 and IgG3 antibodies against *Wb*TSP LEL was measured in the sera of *O. volvulus* putatively immune (PI) or *O. volvulus* infected individuals (INF) using an indirect ELISA described by MacDonald et al., [31]. Briefly, wells of a microtiter Immunolon 2 plates (Dynex, Chantilly, VA), were coated with 1 µg/ml r*Wb*TSP LEL diluted in 0.05M carbonate-bicarbonate buffer, (pH 9.6) overnight at 4°C. Non-specific sites were blocked with 3% BSA in PBS-T for 2 h at 37°C. After washing the plates six times with PBS-T, individual serum samples that were pre-cleared with *E. coli* lysate (400 μg/ml, Promega, Madison, WI) and diluted to 1:100 in binding buffer (1% non-fat milk in PBS-T) were added to each well in duplicate and incubated for 1 h at 37°C. Pre-clarification using *E. coli* lysate was performed as described previously [30] to remove any cross-reacting *E. coli* specific antibodies in the sera samples that may interfere in our assay since all our recombinant proteins were prepared in *E. coli*. Monoclonal antibodies (1:1000 dilution) against different human IgG subclasses (Hybridoma Reagent Laboratory, Kingsville, MD) was used as the secondary antibodies and horseradish peroxidase-conjugated goat anti-mouse IgG (H+L) (KPL, Gaithersburg, MD, 1:8000, incubated for 60 min at room temperature) was used as the detection antibodies. Absorbance (OD_450_) was read after stopping the reaction with an equal volume of 1M H_2_SO_4_ using an E_max_ ELISA reader (Molecular Devices, Sunnyvale, CA). 

We also depleted anti-*Wb*TSP LEL IgG antibodies from the sera of EN subjects by passing the sera through a cobalt IMAC resin column coupled to r*Wb*TSP LEL [35]. Briefly, 1 mg of his-tagged r*Wb*TSP LEL was coupled to 2 ml bed volume of IMAC resin for 2 hrs at 37°C. After washing the resin once with 10 ml of PBS (pH.8), 200 ml of pooled sera was added and incubated overnight at 4°C. After incubation, the resin mixture was centrifuged for 2 min at 750 rpm and the supernatant were collected. Total IgG was removed by incubating sera samples with Protein A gel beads (Thermo Fisher Scientific). Depletion of r*Wb*TSP LEL specific antibodies in the sera samples was confirmed by an indirect ELISA as described above. 

### Antibody dependent cell mediated cytotoxicity (ADCC) assay using human and mouse sera

*In vitro* ADCC assay was performed using both human and mice sera as described previously [34]. Briefly, ten L3 of *B. malayi* were incubated with 2 x 10^5^ peripheral blood mononuclear cells (PBMC) collected from normal healthy subjects, 50 µl of pooled EN sera samples and 50 µl of RPMI 1640 media in a 96 well culture plate (Thermo Fisher Scientific). After 48 h of incubation at 37°C and 5% CO_2_, the larval viability was determined under a light microscope (400 X). Viable larvae were actively moving, coiled and translucent. Dead larvae were flaccid, transparent, damaged and had clumps of cells attached to them. ADCC was estimated as percent larval death calculated using the following formula: Number of dead larvae ÷ Total number of larvae x 100. ADCC assay was also performed using sera samples from rTSP LEL immunized mice. 2 x 10^5^ peritoneal exudates cells (PEC) from normal mice were used as effector cells in these assays. 

### Analysis of cross reactivity between lymphatic filarial TSP LELs and *Onchocerca* TSP LEL

Cross reactivity of anti-*Wb*TSP LEL IgG antibodies against *Ov*TSP LEL was evaluated using an indirect ELISA as described above. The cross reactivity was also evaluated between *Bm*TSP LEL, *Wb*TSP LEL and *Ov*TSP LEL using an immunoblot analysis. Briefly, nitrocellulose membrane blotted with the each of the recombinant proteins was probed with sera collected from mice immunized with r*Bm*TSP LEL or r*Wb*TSP LEL for 1hr at room temperature (RT). After washing with PBST, membranes were incubated for 1hr at room temperature with a secondary goat anti-mouse IgG antibodies conjugated to HRP (1:5000, Thermo Fisher). Color was developed using diamino benzidine (Thermo Fisher Scientific) substrate.

### Analysis of vaccine induced protection in mice

Two weeks after the final vaccination we performed the *in vivo* challenge experiments by surgically implanting 20 *B. malayi* L3 in a micropore chamber in the peritoneal cavity of each mouse [36,37]. After 48 hours, mice were sacrificed and the chambers were surgically removed and examined under the microscope to determine the larval viability. Percent larval death was determined as described before [36,37].

### Antigen specific proliferation assay

Antigen specific proliferation of mouse spleen cells were evaluated as described previously [34]. Briefly, 1 x 10^5^ splenocytes were stimulated with (a) 1 µg/ml of r*Wb*TSP LEL or 1 µg/ml of r*Bm*TSP LEL, (b) 1 µg/ml of a non-specific recombinant protein (recombinant *S. mansoni* G-binding factor, rSmGBF), (c) 1 µg/ml of ConA or (d) medium alone (unstimulated) in triplicate wells for 72hrs at 37°C. Stimulation index (S.I) was calculated from the unstimulated controls. For cytokine analysis, splenocytes were stimulated with 1 µg/ml of r*Wb*TSP LEL and medium alone (unstimulated) for 72 hrs. 

Antigen specific proliferation of PBMC was also determined similarly as described previously [34]. Briefly, PBMC were isolated from heparinized venous blood of study subjects using histopaque 1077 column (Sigma). Isolated PBMC were stimulated with (a) 1 µg/ml of r*Wb*TSP LEL, (b) 1 µg/ml of ConA (positive control), or (c) medium alone (unstimulated controls). PBMC were stimulated in triplicate wells at 37°C in 5% CO_2_ for 72h. Cell proliferation was measured using an MTT assay (Cell Titer 96 R aqueous non-radioactive cell proliferation assay, Promega, Madison, WI). Stimulation indices (S.I) were calculated as above. For cytokine analysis, PBMC were stimulated with 1 µg/ml of r*Wb*TSP LEL and medium alone (unstimulated) for 72 hrs. 

### Analysis of cytokine levels in the culture supernatants of the antigen specific cells

After 72hrs of stimulation with the antigens, culture supernatants of splenocytes were collected and the levels of secreted cytokines (IL-1, IL-4, IL-5, IL-10, IFN-γ, TNF-α) were measured using multianalyte ELISA array kit according to manufacturer’s instructions (SA biosciences, Valencia, CA). Cytokine values in the culture supernatant of cells incubated with rSmGBF or media alone was used as the background value. For human assays, levels of Interferon-γ (IFN-γ), Interleukin-4 (IL-4) and Interleukin- 10 (IL-10) were estimated in the culture supernatants of PBMC 72 hrs after incubation using human cytokine ELISA kits (E-biosciences, San Diego, CA) as per the manufacturer’s instruction. Sensitivity and minimum detection limits of the kits were 0.05 - 0.1 pg/ml. Concentration of each cytokine in the culture supernatant was plotted from a standard curve using recombinant rIFN-γ, rIL-4 or rIL-10 and the data was expressed as pg/ml. 

### Statistical analysis

GraphPad Prism version 5.0 (GraphPad Software, San Diego, CA) was used to analyze the data. Comparisons between two individual data points were made using a Student’s t-test. For multiple comparisons, one way ANOVA was used along with the Tukey–Kramer and/or Dunnet’s post-test wherever appropriate. For cytokine proliferation two-way ANOVA was used with Bonferroni post test. A probability (P) value of <0.05 was considered statistically significant. 

## Results

### *Bm*TSP LEL is expressed on the surface of *B. malayi* L3.

**Figure 1:**
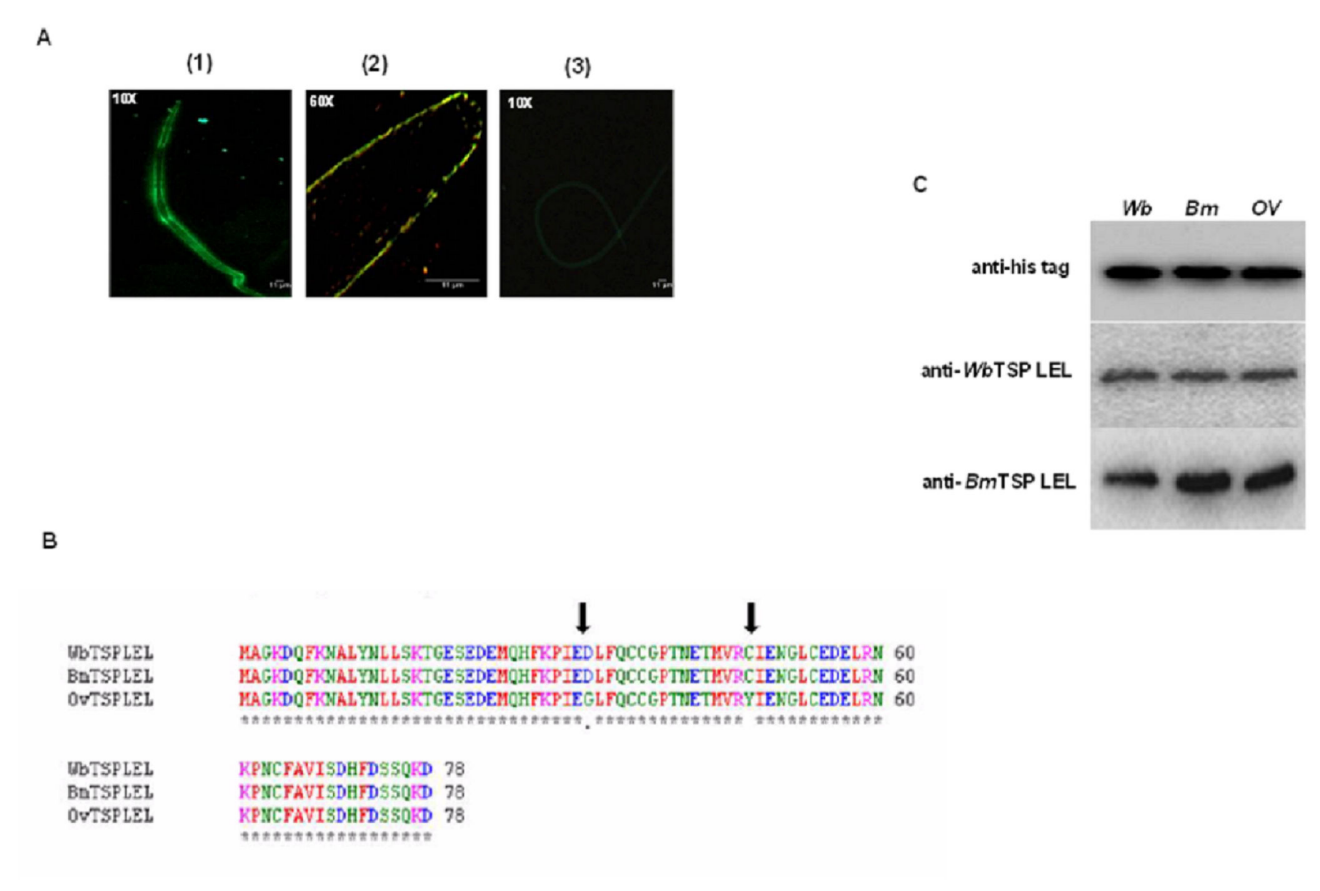
Characterizations of TSP LEL. A. *Bm*TSP LEL is expressed on the cuticle of *B. malayi* L3. Presence of *Bm*TSP LEL on the surface of *B. malayi* L3 was demonstrated by staining L3 with mouse anti-*Bm*TSP LEL antibodies followed by FITC conjugated anti-mouse IgG (1,2). Green fluorescence denotes regions where antibody was bound. No fluorescence found when L3 was stained with control negative sera (3). Scale bar is 11µm. B. Multiple sequence alignment of Tetraspanin was performed using clustalW online tools. Sequence alignment showed that LEL domain of *Wb*TSP is 100% similar to *Bm*TSP and 97% similar to *Ov*TSP. Arrow showing only two amino acids (C_35_-Y_35_ /D_48_ -G_48_) of *Ov*TSP LEL was different from *Bm*TSP LEL and/or *Wb*TSP LEL. C. Immunoblot was performed by probing anti-His tag, anti-*Bm*TSPLEL or anti-*Wb*TSPLEL antibodies. Both anti-*Wb*TSPLEL and anti-*Bm*TSP LEL antibodies cross reacted with r*Ov*TSP LEL proteins.

 Recombinant *Wb*TSP LEL, r*Bm*TSP LEL and r*Ov*TSP LEL with his tag had same molecular size of 12 kDa and appeared as a single band in the SDS PAGE gel. The predicted size of *Bm*TSP LEL is 8 kDa plus his-tag resulted in 12 kDa. Subsequently we immunized mice with r*Bm*TSP LEL and generated polyclonal antibodies. TSP is one of the relatively few integral membrane proteins that are consistently found in the tegument of *S. mansoni* [13], where they are believed to play an important role in cellular interactions and help maintain the integrity of tegument membrane [16,38]. Therefore, we first wanted to determine if *Bm*TSP LEL is expressed on the surface of the third stage larvae (L3) of *B. malayi*. Our results showed significant fluorescence uniformly present throughout the cuticle of L3 (Figure 1A). These findings suggested that *Bm*TSP LEL is expressed on the surface of L3. Incubation with control sera or secondary antibodies alone did not show any fluorescence on the surface of L3 (data not shown).

### Anti-*Bm and Wb* TSP LEL cross react with *Ov* TSP LEL.

Sequence alignment of *Wb*TSP LEL showed 100% and 97% homology with *Bm*TSP LEL and *Ov*TSP LEL respectively (Figure 1B). This was further confirmed by comparing the sequence of TSP LEL with published *Wb*TSP (Accession No: WUBG_09786) sequence at the NCBI site. This analysis showed 10aa differences in the LEL regions (data not shown). Polymorphisms have been reported in the *SmTSP-2* gene as well [39]. Thus, we believe that these differences are due to polymorphisms in the TSP proteins, rather than to species differences. Subsequently, we also performed an immunoblot analysis to check the cross reactivity of anti-*Bm*TSP LEL and anti-*Wb*TSP LEL antibodies with r*Ov*TSP LEL. Results showed that anti–*Bm*TSP LEL and anti-*Wb*TSP LEL antibodies cross react with r*Ov*TSP LEL (Figure 1C).

### Both LF human and mice sera carry cytophillic antibodies against TSP LEL

**Figure 2:**
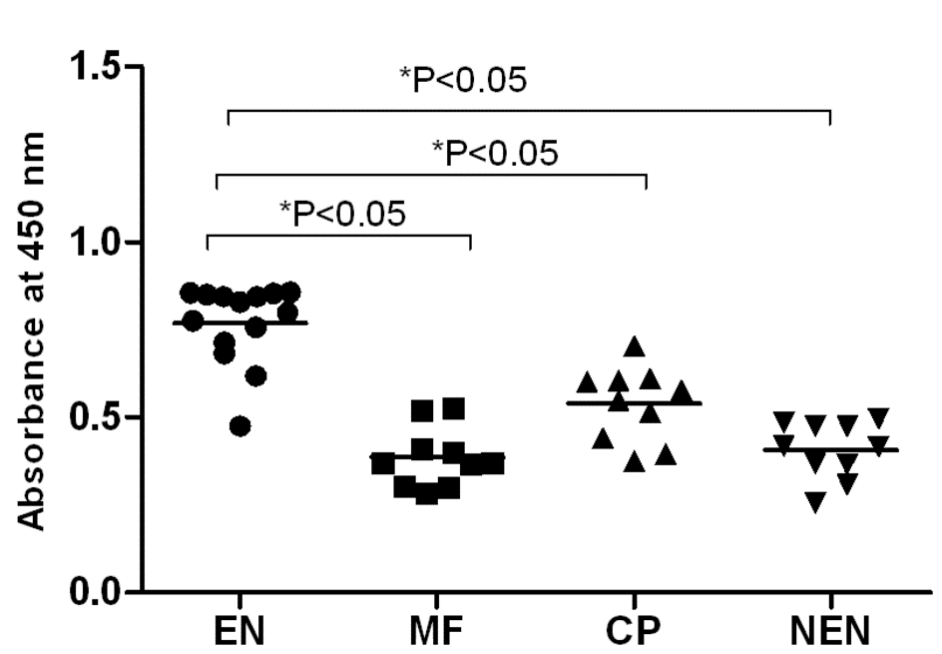
TSP LEL specific IgG antibodies in the sera of LF human subjects. Levels of total IgG antibodies against r*Wb*TSP LEL in the sera of Endemic normal (EN), Microfilaremic (MF), Chronic pathology (CP) and Non-endemic normal (NEN) subjects were measured using an indirect ELISA. 15 sera samples (diluted 1:100) were evaluated from EN, and 10 samples from MF, CP and NEN individuals. Each data point represents sera sample from a single individual. Horizontal lines represent geometric mean value. Data is represented as scatter plot where each dot represents absorbance of individual sera. Significant (*P<0.05) IgG antibodies in EN individuals compared to other groups (One way ANOVA along with Tukey-Kramer post statistics test was used).

**Figure 3:**
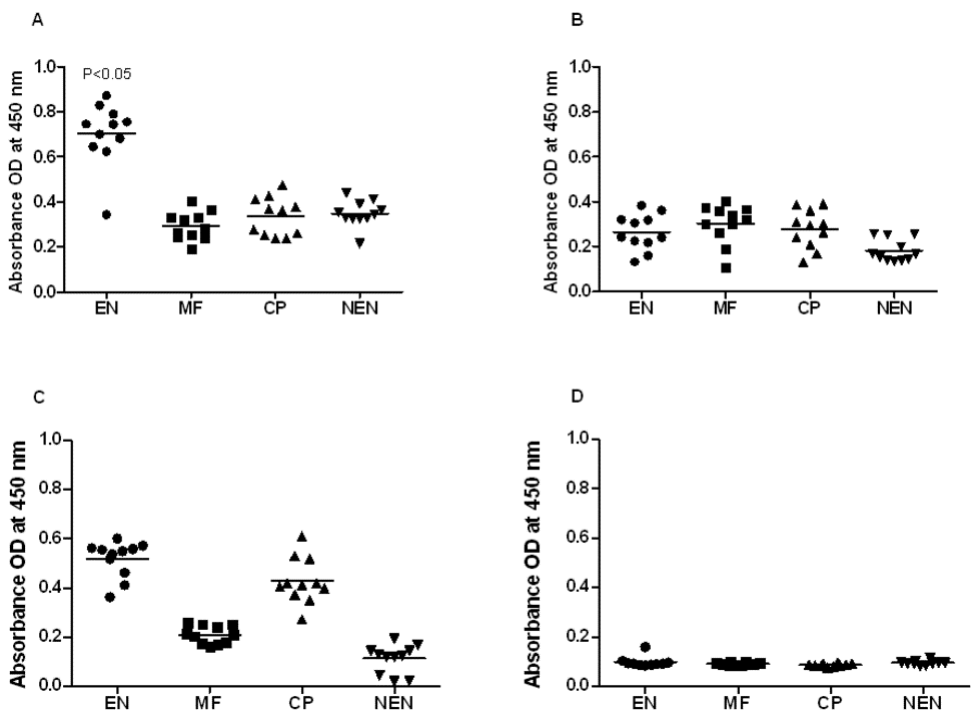
TSP LEL specific IgG isotype of antibodies in the sera of human. Isotype of IgG antibodies A) IgG1, B) IgG2, C) IgG3 and D) IgG4 against r*Wb*TSP LEL were measured in the sera of putatively immune individuals (n=10) using an indirect ELISA. Each data point represents sera sample from a single individual. Horizontal lines represent geometric mean value. Data is represented as scatter plot where each dot represents absorbance of individual sera. Significant *(P<0.05) levels of isotype antibodies in EN individuals compared to other groups (One way ANOVA along with Tukey-Kramer post statistics test was used).

Expression of TSP LEL on the surface of *B. malayi* makes the antigen easily available to the host immune system. To determine if the sera from putatively immune and infected individuals carry antibodies against filarial TSP LEL, we first screened for the presence of anti-TSP LEL antibodies in the sera of different subjects (EN, CP, MF and NEN) using an indirect ELISA. Our results show that 14 out of 15 putatively immune endemic normal (EN) individuals carry circulating IgG antibodies against r*Wb*TSP LEL (Figure 2) proteins. No significant levels of IgG were observed in the sera samples of MF, CP and NEN individuals. Positive reactions in the EN sera were determined as readings above the cut off value of mean + 3SD of NEN OD values. We also analyzed the isotype of anti-*Wb*TSP LEL IgG antibodies in these sera samples. Our results showed that anti-TSP LEL IgG1 were predominant in the sera of EN individuals (Figure 3) compared to sera from other filarial groups (MF and CP). High levels of IgG3 antibodies were found both in EN and CP individuals. IgG2 and IgG4 isotypes did not show any significant differences between the filarial groups. Anti-TSP LEL IgG antibodies were not detectable in the sera of NEN individuals. Similar antibody responses were observed against r*Bm*TSP LEL (data not shown).

**Figure 4:**
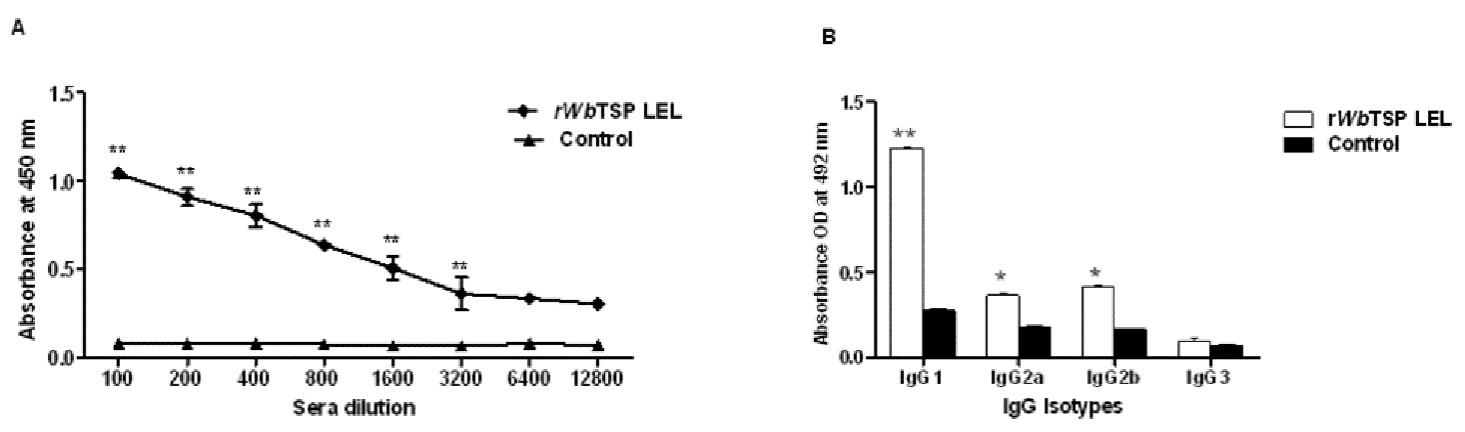
TSP LEL specific IgG antibodies in the sera of mice. Mice were immunized four times at two weeks interval using 15 µg of r*Wb*TSP LEL combined with alum adjuvant. **A**) **Titer of anti-TSP LEL IgG antibodies**. Approximately, 100 ng of recombinant proteins (100 ng /100µl /well) were coated onto the wells of an ELISA plate and bound serum IgG was detected using an HRP-labeled anti-mouse IgG secondary antibody. Each data point indicates mean ±S.D value from five animals. **B**). **Isotype of anti-TSP LEL IgG antibodies in the sera of mice**. Isotypes specific ELISA was performed as described in the methods section. Bars represent mean ±SD from five mice per group. ** Significant (P<0.001) and * (P<0.05) compared to control groups (Student’s t-test).

Similar results were obtained when we analyzed sera samples from mice immunized with r*Wb*TSP LEL (Figure 4). Immunized mice showed significantly high titers of anti-*Wb*TSP LEL IgG antibodies in their sera (Figure 4A). Subsequent isotype analysis showed that the anti-*Wb*TSP LEL antibodies were predominantly of IgG1, followed by IgG2a and IgG2b isotypes (Figure 4B). Control animals did not show any IgG specific antibodies against r*Wb*TSP LEL. Similar results were observed when r*Bm*TSP LEL was used (data not shown)

### Anti-TSP LEL antibodies participated in the killing of *B. malayi* L3 in an ADCC mechanism

**Figure 5:**
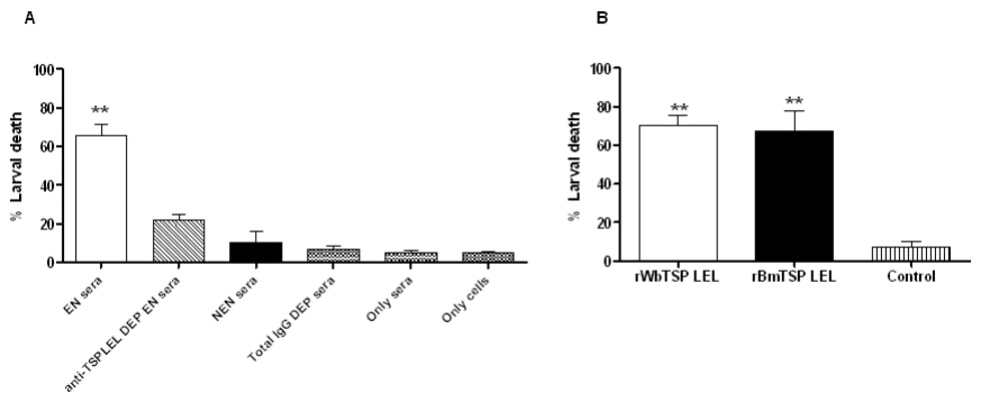
Sera from EN subjects and mice immunized with r*Wb*TSP LEL participated in the killing of *B. malayi* L3 in an ADCC mechanism. **A**) 50 µl of pooled EN sera (n = 10) samples or sera samples depleted off anti-TSP LEL antibodies were incubated with 2x10^5^ peripheral blood mononuclear cells from normal healthy individuals and 10 *B. malayi* L3 for 48 hrs at 37°C. **B**) 50 µl of pooled sera (n = 5) samples from r*Wb*TSP LEL immunized or r*Bm*TSP LEL immunized mice were incubated with 2x10^5^ peritoneal exudates cells from naive mice and 10 *B. malayi* L3 for 48 hrs at 37°C. Larval viability was evaluated at the end of the incubation. Values represent mean ± SD of three wells. Significant larval death ** (P<0.001) compare to controls analyzed by Student’s t-test.

Results presented above show that sera of EN subjects and immunized mice have significantly high titer of anti-TSP LEL IgG antibodies. IgG antibodies can bind to FcγRI and FcγRII receptors expressed on several inflammatory cells such as macrophages, neutrophils, eosinophils and participate in an ADCC mechanism [40]. Therefore, in this study we evaluated if anti-TSP LEL antibodies in the sera of EN subjects and immunized mice can participate in the killing of *B. malayi* L3 *in vitro* in an ADCC assay. Our results show that pooled sera samples from EN subjects were able to kill 65% of *B. malayi* L3 in an ADCC assay. Depletion of anti-*Wb*TSP-LEL antibodies from the sera of EN subjects resulted in the reduction of the killing ability of the sera by 2/3. When we depleted total IgG from the sera of EN subjects the killing ability was reduced by 85%. Incubating the larvae with cells alone or sera alone killed only <5% of L3 (Figure 5A). A similar ADCC assay performed using sera samples from immunized mice showed that ~67% of *B. malayi* L3 were killed when immune sera was used, whereas, only 5% of L3 were killed when sera samples from control animals were used (Figure 5B). These findings suggest that the anti-TSP LEL antibodies play a critical role in the killing of *B. malayi* L3 *in vitro*.

### Antibodies in the sera of EN subjects can recognize r*Ov*TSP LEL.

Another important finding in this study was that the IgG antibodies in the sera of EN subjects could recognize r*Ov*TSP LEL. Further analysis showed that the r*Ov*TSP LEL recognizing antibodies in the sera of EN subjects were of IgG1 and IgG3 isotype. This suggested that potentially the same antibodies may be recognizing both r*Wb*TSP LEL and r*Ov*TSP LEL. This was not surprising given the significant sequence similarities between *Ov*TSP LEL and *Wb*TSP LEL.

**Figure 6:**
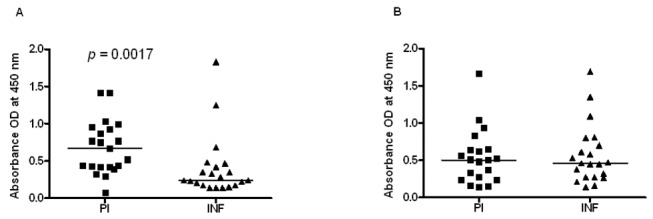
Sera from individuals who are putatively immune against *O. volvulus* infections (PI) has antibodies that cross react with r*Wb*TSP LEL. Isotype of IgG antibody **A**) IgG1 and **B**) IgG3 reactivity was measured in the sera of putatively immune individuals (PI) and *O. volvulus* infected individuals (INF) against r*Wb*TSP LEL. Each data point represents sera sample from a single individual (n=20). Horizontal lines represent median value. Data is represented as scatter plot where each dot represents absorbance of individual sera. PI sera showing significant (P<0.001) cross reactivity of IgG1 antibodies compared to INF individuals. The OD of NEN (NYC individuals) was always below OD 0.1. Significant (P<0.001) levels of isotype antibodies in PI compared to INF group (Student’s t-test).

We then tested the reverse cross reactivity, that is, if antibodies in the sera of Ov-PI subjects (these are PI subjects from an *O. volvulus* endemic area in Cameroon) will react with r*Wb*TSP LEL. Our results show that IgG1 specific antibodies in the sera of Ov-PI individuals recognized r*Wb*TSP LEL proteins (Figure 6A). Levels of anti-*Wb*TSP LEL IgG3 antibodies were not significantly different in the sera of Ov-PI subjects and *O. volvulus* infected patients (Figure 6B). These findings suggested the presence of cross reactive IgG1 antibodies in the sera of LF-EN and Ov-PI subjects. 

### Vaccination of mice with rTSP LEL conferred protection against a challenge infection.

Vaccine potential of r*Bm*TSP LEL or r*Wb*TSP LEL was confirmed in the mouse model by challenging with 20 *B. malayi* L3 in a micropore chamber. Mice vaccinated with r*Bm*TSP LEL and/or r*Wb*TSP LEL gave 65% and 67% protection respectively against *B. malayi* L3 challenge (Table 1).

**Table 1:** Vaccine induced protection in mice.

Animal groups	Number of L3 recovered Mean ± S.D	Number of Dead L3 Mean ± S.D	% Larval death Mean ± S.D
**Trial-I**			
Alum (Control)	16±1.34	1.4±0.543	8.27±2.592
r*Wb*TSPLEL	18±2.70	11.6±2.073	63.2±7.505**
r*Bm*TSP LEL	16±2.70	10.6±0.894	65.44±6.898**
**Trial-II**			
Alum (Control)	19.2±2.38	2.2±0.83	2.2±0.83
r*Wb*TSPLEL	15±0.707	9.75±1.14	64.28±9.49**
r*Bm*TSP LEL	17.25±2.79	10.25±1.67	60.33±3.46**

In vivo micropore chamber assay was performed by surgically implanting 20 B. malayi L3 into the peritoneal cavity of each mouse. 48 hrs after implantation, chambers were removed and larval viability and larval death was determined. Values are mean ± SD. N = 5. Data presented here is from one of two similar experiments showing comparable results.

** significant larval death (P < 0.001) compared to control mice groups analyzed by one way ANOVA followed by Dunnett’s post ANOVA test.

rTSP LEL specific IFN-γ secreting cells were present in the PBMC of EN subjects and in vaccinated mice**.**

**Figure 7:**
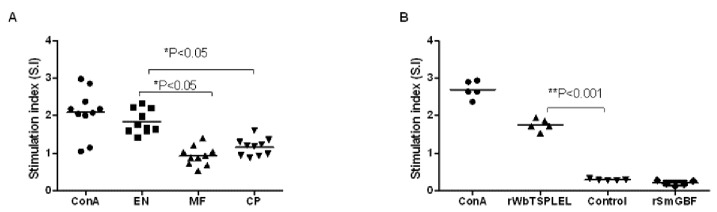
Proliferation of PBMC from human subjects and spleen cells from vaccinated mice following stimulation with r*Wb*TSP LEL. **A**. PBMC from Endemic Normal (EN), Microfilaraemic (MF) and Chronic Pathology (CP) groups were stimulated with r*Wb*TSP LEL. Concanavalin A (ConA) was used as a positive control. Significantly high S.I. *(P<0.05) for cells from EN compared to cells collected from other groups, analyzed by two way ANOVA with Bonferonni test. **B**. Splenocytes from mice immunized with r*Wb*TSP LEL and alum proliferated significantly to r*Wb*TSP LEL stimulation. ConA was used as a positive control. Non-specific recombinant protein (recombinant *S. mansoni* G-binding factor, rSmGBF) was used as a negative control. Each data point represents stimulation index (S.I) of individual human sample (n = 10) and mice (n=5). Significantly high S.I. **(P<0.001) for cells from vaccinated animals compared to control cells analyzed by student’s t-test.

PBMC from putatively immune EN subjects and spleen cells from vaccinated mice proliferated significantly in response to r*Wb*TSP LEL stimulation suggesting the presence of antigen-specific memory cell population in both human and mice (Figure 7A and 7B). The stimulation indices were 1 fold and 2 folds higher compared to NEN individuals and control mice respectively. PBMC from MF and CP individuals did not show any significant proliferations. Similarly, spleen cells from control mice also did not show any significant proliferation.

**Figure 8:**
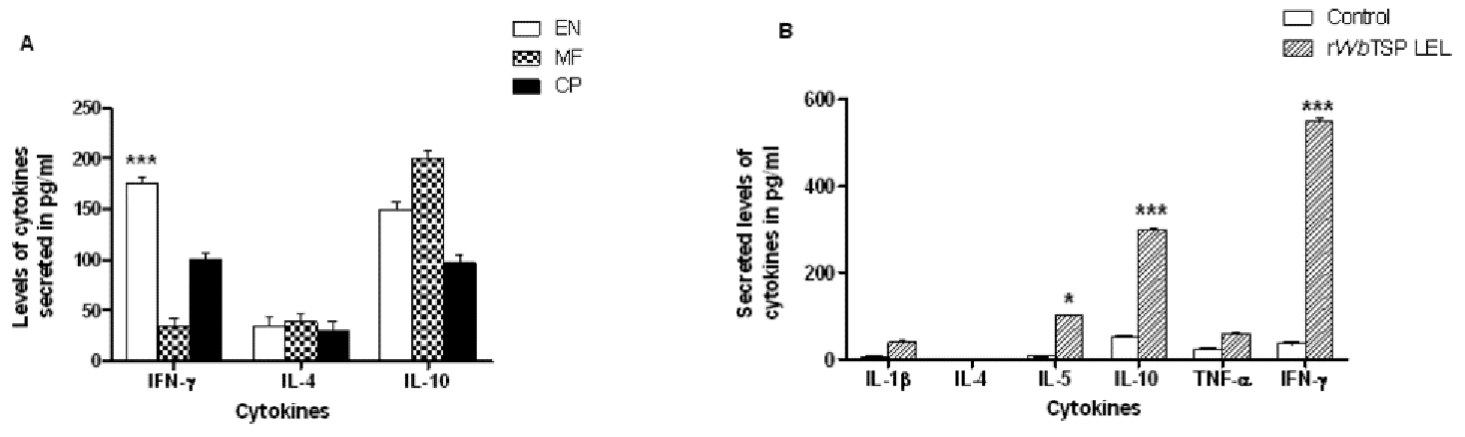
Cytokine responses of r*Wb*TSP LEL responding cells in the PBMC of human subjects and in the spleen of vaccinated mice. Cytokines (pg/ml) in the culture supernatants of human PBMC and mice spelnocytes were measured using an ELISA. **A**) Results show that significant level of IFN-γ is secreted by PBMCs from EN subjects in response to r*Wb*TSP LEL. Significant ***(P<0.001) compared to other two groups (CP and MF) analyzed by One way ANOVA along with Tukey-Kramer post statistics test. **B**). IFN-γ level was prominent in the culture supernatants of spleen cells from r*Wb*TSP LEL immunized mice compared to controls. Levels of secreted IL-10 were also high in the culture supernatants of EN PBMC and in the spleen cell culture supernatants of immunized mice. The data shown are normalized with the unstimulated controls. Significant cytokine levels ***(P<0.001) *(P<0.05) compared to control groups analyzed by Student’s t -test. Data is from one of two similar experiments showing comparable results.

Analysis of the levels of secreted cytokines in the culture supernatants of PBMC stimulated with r*Wb*TSP LEL showed that significantly high levels of IFN-γ (168 pg/ml) (P<0.001) was secreted by PBMC from EN subjects, whereas, PBMC from MF individuals secreted predominantly IL-10 (210 pg/ml) (Figure 8A). There were no significant differences in the levels of IL-4 in the culture supernatants of PBMC from different groups. Splenocytes from mice immunized with r*Wb*TSP LEL secreted significant (P<0.05) amounts of IFN-γ (550 pg/ml) and IL-10 (300 pg/ml) in response to r*Wb*TSP LEL (Figure 8B). However, secreted levels of IL-1, TNF-α, IL-4 and IL-5 were not significantly different in the culture supernatants of splenocytes compared to control.

## Discussion

Lymphatic filariasis is the second leading cause of disability in the world [41], and is thus considered a major obstacle to economic development in the developing countries where this disease is endemic. Mass drug administration sphere headed by the World Health Organization is making substantial headway in reducing the incidence of this infection in several parts of the world [4]. Additional strategies such as vector control measures and an effective vaccine to prevent lymphatic filariasis will substantially supplement the effort towards elimination of this disease. Several previous studies have shown the feasibility of developing a vaccine against lymphatic filariasis and a number of potential antigens were identified as suitable candidates for vaccine against this infection in experimental animals [5, 7-9, 42]. In this study we show that the large extracellular loop of tetraspanin (TSP LEL) is a potential vaccine candidate against lymphatic filariasis. We also show that TSP LEL is present in several filarial parasites and antibodies generated against TSP LEL from one filarial parasite can cross-react with TSP LEL antigen from another filarial parasite suggesting a broader potential for developing TSP LEL as a common vaccine against several filarial parasites.

Molecules expressed on the surface of the parasite tend to modulate or down regulate host immune responses so the parasite can successfully establish infection in its host and/or escape host detection [43]. Targeting these parasite surface molecules have been used as a control strategy to reverse the host immunomodulation and allow the host immune system to recognize the parasite and eliminate them form the host [12]. Previous studies showed that TSP is expressed on the surface of helminthes [44,45]. Surface staining with anti-TSP LEL antibodies confirm that *Bm*TSP LEL is also expressed on the surface of *B. malayi* L3. We have already reported that *Bm*TSP is present in *B. malayi* L3, adults and Mf stages [21]. Thus, all stages of the parasite may potentially express TSP LEL suggesting an important role for this protein in these parasites. Targeting TSP by developing a vaccine against TSP LEL afforded substantial protection against primary alveolar echinococcosis [46] and *S. mansoni* [12]. These reports suggested that TSP LEL is an attractive target for developing a vaccine against helminthes.

Our previous studies showed that differential recognition of protective filarial antigens by endemic normal individuals can be a useful and rigorous approach to identify vaccine candidates against filarial infections [47]. Several previous studies demonstrate the presence of high titer of antibodies against key antigens in the sera of putatively immune endemic normal individuals [48,49]. *In vitro* ADCC assays confirm that these antibodies can participate in the killing of infective larvae of filarial parasites. Presence of these host protective antibodies in EN subjects, but not in the infected individuals (MF, CP) potentially suggests a strong correlation for selecting the candidate antigen that elicited the protective antibodies for further testing as a vaccine candidate. Based on this paradigm, in this study we first analyzed if anti-TSP LEL antibodies are uniquely present in EN subjects. Our results confirm the presence of high titer of anti-TSP LEL IgG antibodies in the putative immune EN subjects. These anti-TSP LEL IgG antibodies were found to be primarily cytophilic antibodies (IgG1 and IgG3). Similar antibody responses (IgG1 and IgG3) were demonstrated against *Sm*TSP-2 LEL as well in individuals who are putatively resistant against schistosomiasis [12]. These findings suggest that parasite TSP LEL in general induces similar antibody responses in human. No detectable levels of r*Wb*TSP LEL specific IgE antibodies were present in the sera of EN or in the sera of subjects who have filarial infection (MF and CP) (data not shown). This suggests that if r*Wb*TSP LEL were to be developed as a vaccine for human use, potential allergic reactions will be minimal. 

Immunization of mice with r*Bm*TSP LEL or r*Wb*TSP LEL resulted in the generation of anti-TSP LEL IgG1, IgG2a and IgG2b antibodies similar to that reported in mice vaccinated with *Sm*-TSP-2 [12]. These findings also suggested that parasite TSP LELs potentially induces similar types of antibody responses in the mouse model. Immune human subjects carry anti-TSP LEL IgG1 and IgG3 antibodies which is equivalent to IgG2a and IgG2b in mouse.

IgG1 and IgG3 antibodies can mediate antibody-dependent cellular cytotoxicity (ADCC) [50] and phagocytosis [51] by a variety of effector cells that express FcγRI and FcγRIII. Elevated levels of these cytophilic antibodies against r*Wb*TSP LEL in putatively immune EN subjects and immunized mice suggested that these antibodies may participate in the killing of the parasite via ADCC mechanism. In fact, our results confirm that the anti-*Wb*TSP LEL antibodies in LF-EN subjects and immunized mice have the ability to participate in the killing of *B. malayi* L3 through an ADCC mechanism. When we depleted the anti-*Wb*TSP LEL antibodies from the sera samples, ability of the sera samples to participate in the killing *B. malayi* L3 was substantially decreased suggesting a key role for the anti-*Wb*TSP LEL antibodies in the killing mechanism. These findings suggested that the anti-*Wb*TSP LEL antibodies may have an important role in the defense against this parasitic infection in human.

Subsequently we evaluated the vaccine potential of r*Wb*TSP LEL in the mouse model using a micropore chamber challenge approach. Although this is not truly a challenge model, given the difficulty in performing immunological analysis in jirds and due to prohibitive cost for the primate trials, the mouse micropore challenge system was used as a surrogate to challenge studies. Nevertheless, the data was interpreted with caution. Our results showed that immunization with r*Bm*TSP LEL or r*Wb*TSP LEL conferred 65% larval killing in this model. Compared to other vaccine proteins (*Bm*HSP12.6, *Bm*GST, *Bm*VAL, *Bm*TPX-2) tested in our laboratory [25][33] [35][42], r*Bm*TSP LEL or r*Wb*TSP LEL appears to be a good vaccine candidate against *B. malayi*. We also observed several cells attached firmly to the dead larvae in the micropore chambers. We did not identify the morphology of these cells; however, based on our ADCC results, we believe that these are possibly the effector cells. Protection conferred by r*Bm*TSP LEL or r*Wb*TSPLEL is also comparable to the protection reported for *Sm*TSP-2 (57%–64%) in mice [12]. Similarly, vaccination trials in cestode infections also suggest that TSP is an attractive vaccine candidate against helminthes [46].

Evaluation of the immune correlates of r*Bm*TSP LEL-induced protection showed that antigen specific memory cells are present in putatively immune LF-EN subjects and in vaccinated mice. Analysis of the cytokines secreted by these antigen-specific proliferating cells revealed that both PBMC from immune individuals and vaccinated mice splenocytes secreted IFN-γ and IL-10 in response to rTSP LEL stimulation. These findings were similar to those reported after vaccination of mice with *Sm*TSP-2 [52]. Presence of antigen-specific IFN-γ secreting cells together with IL-10 secreting cells in the spleen suggest a balanced Th1/Th2 immune responses that may be important for regulating the inflammation caused by the host-protective mechanism in the filarial infections or for preventing the development of highly polarized Th1 responses [53,54]. Increase in the levels of IFN-γ suggests the activation of macrophages that may be important in the ADCC mediated killing of parasites observed in our studies [35] and others [55].

Another important observation in our studies is the finding that the TSP LEL sequences from different filarial parasites (*B. malayi, W. bancrofti, O. volvulus*) share sequence identity. This was reflected in the anti-TSP LEL antibody cross reactivity observed in the sera of putatively immune individuals for lymphatic filariasis and onchocerciasis. Because of the significance sequence identity, filarial TSP LEL can be used as a cross-protective vaccine antigen against both lymphatic filariasis and possibly *O. volvulus* infections in human. This observation has broader implications for the control of both the infections, especially, in parts of Africa where both these infections occur as co-infections [4]. However, challenge studies in animals are required to confirm this. Recently there is significant interest in identifying cross-protective vaccine antigens that are effective against multiple closely-related pathogens in an effort to reduce the cost of manufacturing and development pipelines [56]. 

In summary, we show that TSP LEL is expressed on the surface of *B. malayi* L3 and are thus easily accessible to the host immune system. The fact that putatively immune individuals carry IgG1 and IgG3 antibodies against *Wb*TSP LEL and these antibodies participate in the killing of L3 in an ADCC assay confirm the potential of this antigen as a vaccine candidate against lymphatic filariasis. Our vaccination trials in the mouse model confirm this notion. Significant cross-reactivity of the anti-*Wb*TSP LEL IgG1 antibodies in the sera of individuals who are protected against both lymphatic filarial and onchocerciasis infections suggest that *Wb*TSP LEL could be developed as a potential vaccine candidate against onchocerciasis. These are only preliminary observations additional trials are needed to confirm the vaccine potential of rTSP LEL against filarial infections.
